# Silicon Wafer Functionalization with a Luminescent Tb(III) Coordination Complex: Synthesis, Characterization, and Application to the Optical Detection of NO in the Gas Phase

**DOI:** 10.3390/molecules24101914

**Published:** 2019-05-17

**Authors:** Bijal K. Bahuleyan, Kathleen Toussaint, Hervé Rinnert, Raphaël Vallon, Michaël Molinari, Françoise Chuburu, Cyril Cadiou

**Affiliations:** 1 Department of General Studies, Yanbu Industrial College, Yanbu Al Sinaiyah 30799, Saudi Arabia; 2 Institut de Chimie Moléculaire de Reims, CNRS UMR 7312, Université de Reims Champagne-Ardenne URCA, 51687 Reims CEDEX 2, France; kathleen.toussaint@univ-lorraine.fr (K.T.); francoise.chuburu@univ-reims.fr (F.C.); 3 Institut Jean Lamour, CNRS UMR 7198, Université de Lorraine, 54506 Vandœuvre-lès-Nancy CEDEX, France; herve.rinnert@univ-lorraine.fr; 4 Laboratoire de Recherche en Nanosciences, EA 4682, Université de Reims Champagne-Ardenne URCA, 51685 Reims CEDEX 2, France; michael.molinari@u-bordeaux.fr; 5 Groupe de Spectrométrie Moléculaire et Atmosphérique, CNRS UMR 7331, Université de Reims Champagne-Ardenne URCA, 51685 Reims CEDEX 2, France; raphael.vallon@univ-reims.fr; 6 Institut de Chimie et Biologie des Membranes et des Nano-objets, CNRS UMR 5248, University of Bordeaux, IPB, 33600 Pessac, France

**Keywords:** lanthanide complexes, silicon wafers, DOTAGA ligand, optical properties, NO detection

## Abstract

A new luminescent Tb-DOTAGA (1,4,7,10-tetraazacyclododecane-1-glutaric-4,7,10- triacetic acid) complex (TbL) was synthesized and covalently immobilized on a silicon wafer. The grafting process was monitored by means of IR and XPS spectroscopies and the optical properties of the functionalized silicon wafer (TbL@Si) were investigated by fluorescence experiments. A homemade setup was then implemented in order to follow TbL@Si optical properties in the presence of gaseous nitric oxide (NO). The prima facie results indicated that in the presence of NO, the wafer fluorescence was partially quenched. This quenching was reversible as soon as NO was pumped outside the fluorescence cell, which could be interesting for the further development of lanthanide labelled silicon wafers as gas phase sensors.

## 1. Introduction

Search for effective strategies to develop chemical sensors has become a great concern for scientists in the past few years. Among the issues needing to be addressed, elaborating sensors for chemical species in the vapor phase for environmental emission control, clinical assaying, and explosive detection are of the upmost importance [1]. Especially, selective detection of nitric oxides (NOx) gases has become one of the most challenging areas concerning environmental effects and biological applications. NO is not only an atmospheric pollutant but also a messenger in the cardiovascular, nervous, and immune system [2,3]. For NO sensing, a wide variety of methods have been reported involving electrochemical, electrical resistance, X-ray photoelectron spectroscopy, electron paramagnetic resonance spectroscopy, colorimetric, fluorometric, and chemiluminescent detection [2,3,4,5,6,7,8,9,10,11]. Among these, fluorometric methods using luminescent probes are promising in terms of sensitivity, selectivity, and experimental feasibility [12]. To act as the luminescent probe, lanthanide (Ln^3+^) ions are well-adapted candidates because they have sharp emission peaks, very large Stokes shifts, and long luminescence lifetimes which give a unique characteristic to effectively eliminate short-lived fluorescence from the surroundings of the lanthanide ion [13]. The two main sensing approaches that are described in the literature are Ln^3+^ ions which can either be encapsulated within supramolecular structures (supramolecular-based sensing approach), and chelated within a molecular framework (molecular-based sensing approach) [14]. To illustrate the first strategy, Ln^3+^ ions have been recently incorporated within porous metal-organic frameworks (MOFs). These materials constitute an excellent platform for host-guest chemistry in solution which allows detection by luminescence iron ions [15], copper ions and small molecules [16] or nitroaromatic explosives [17]. For the second strategy, lanthanide chelates, based on polyaminocarboxylate ligands, have been developed to detect in solution by time-gated luminescence, hypochlorous acid [18] or NO [19]. In all those examples, a fluorescence quenching of the probe is observed in solution when the analyte is present [20,21].

**Scheme 1:**
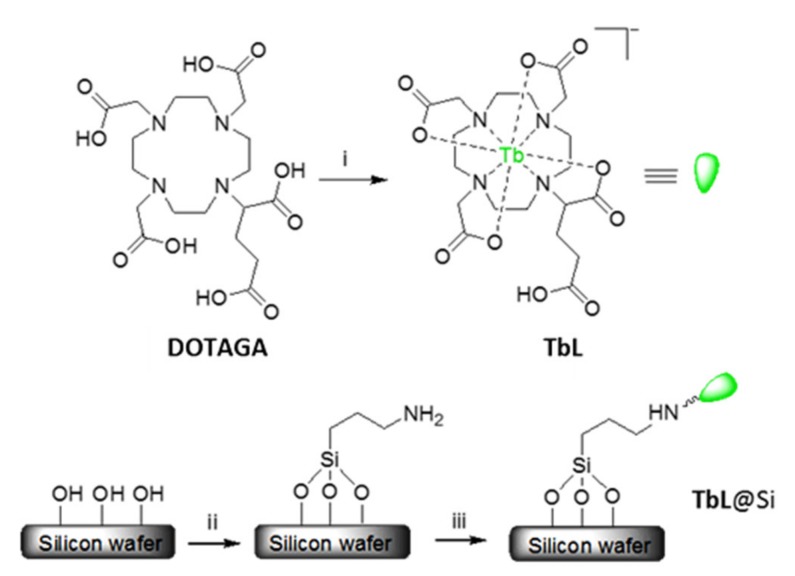
Synthesis and grafting of Tb-DOTAGA complex (TbL) on Si wafer (TbL@Si), (i) Tb_2_O_3_/H_2_O, (ii) (3-Aminopropyl)triethoxysilane (APTES), toluene, (iii) O-(Benzotriazol-1-yl)-N,N,N′,N′-tetramethyluronium tetrafluoroborate (TBTU), N,N-Diisopropyléthylamine (DIPEA), N,N-Diméthylformamide (DMF).

On the contrary, similar studies for the detection of species in the gas phase are very scarce. To our knowledge, only two examples have been described (i.e., a Tb porous coordination polymer was recently used to sense vapours of small molecules [22] whereas Tb-MOF films were able to detect dioxygen versus dinitrogen [23]). In this paper, our purpose is to modify flat silicon wafers with luminescent Tb coordination complexes in order to test if the luminescence of the hybrid material is affected by the presence of chemical species in the gas phase. To achieve this goal, a luminescent Tb-DOTAGA complex (TbL) was anchored on flat oxidized silicon wafers (TbL@Si) (Scheme 1). The choice of silicon wafer was guided by the potential translation of the setup to standard micro- and optoelectronics. This optical device was then implemented and tested in a gas chamber to evaluate its sensitivity to NO. Herein, we describe the design and synthesis of the Tb-DOTAGA complex, its immobilization on silicon wafers, and first evidence for detection of gaseous nitric oxide.

## 2. Results and Discussion

### 2.1. Tb-DOTAGA Complex Synthesis and Spectroscopic Characterization

In order to ensure a high stability for Tb(III) in the macrocyclic cavity and then to avoid its leaching during the grafting process, DOTAGA ligand was chosen as the chelator [24,25]. Indeed, DOTAGA exhibited five carboxylic functions, four dedicated (with nitrogen atoms) to the Tb(III) coordination and the stability of TbL complex, the fifth one remaining available for TbL covalent grafting onto the amine-modified silica surface. From a synthesis point of view, TbL complex was obtained by the addition of Tb_2_O_3_ to an aqueous solution of the DOTAGA ligand. After purification, TbL was recovered in 80% yield (Scheme 1). Tb(III) complexation was demonstrated using IR spectroscopy (Figure S1), by the weakening of the carbonyl vibration from 1715 cm^−1^ for DOTAGA to 1594 cm^−1^ for TbL, as expected for a carboxylate coordination to the metal cation [26]. Moreover, the TbL complex exhibited an additional vibration at 1724 cm^−1^ confirming the presence of the free COOH group, uncoordinated to the metal. TbL characterization was completed using high resolution mass spectrometry study (electrospray ionization) which proves the unambiguous isolation of the complex (Figure S2).

**Figure 1:**
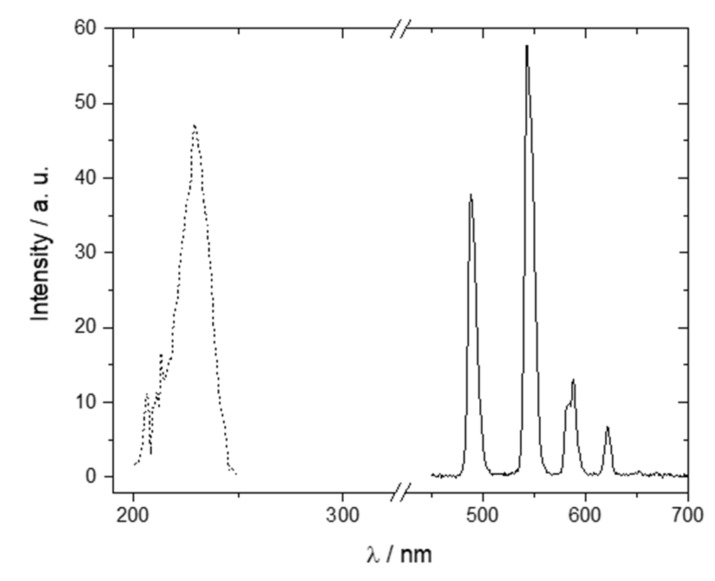
Excitation (dotted line, λ_em_ = 545 nm) and emission fluorescence spectra of TbL in the solid state (298K, λ_exc_ = 230 nm, slit width 5 nm).

Solid state luminescence spectrum of TbL recorded at room temperature (Figure 1) exhibited the four characteristic Tb(III) emission bands in the range 450–650 nm (after excitation at 230 nm) [13]. The narrow emission bands resulted from the radiative transitions between the intra 4f energy levels of the Tb(III) ions. The most intense peak at 545 nm corresponded to the ^5^D_4_–^7^F_5_ transition. The other observed transitions were ^5^D_4_–^7^F_6_ (490 nm), ^5^D_4_–^7^F_4_ (585 nm), and ^5^D_4_–^7^F_3_ (622 nm). Solid state **TbL** fluorescence lifetime measured from the ^5^D_4_ to ^7^F_5_ transition was equal to 2.20 ms as expected for Tb(III) [27]. In solution, TbL fluorescence lifetimes were equal to 2.09 ms in H_2_O and 3.58 ms in D_2_O, indicating the coordination of a single water molecule (q = 0.96, Figure S3) [28].

### 2.2. Functionalization and Characterization of the Silicon Wafers

**Figure 2:**
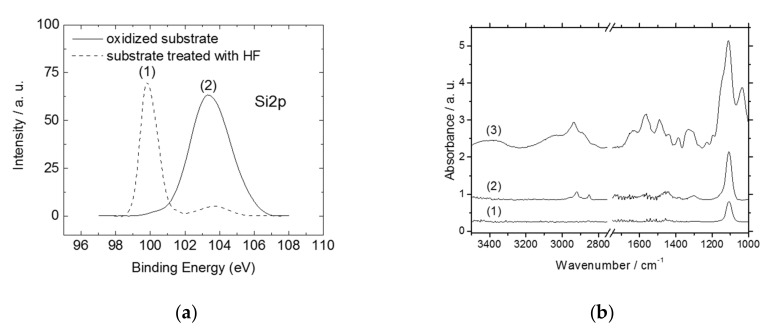
(**a**) Si_2p_ XPS spectrum of the silicon substrate treated with HF (1) and (**b**) after UV/O_3_ oxidation (2). IR absorption spectrum of (1) oxidized Si wafer, (2) wafer after treatment with APTES, (3) TbL@Si wafer.

In order to grow a reproducible SiO_2_ layer at the silicon surface with a well-mastered thickness, silicon wafers were treated beforehand with HF in order to get an almost pure silicon surface. This was controlled in the XPS Si2p spectrum by the presence of an intense band around 99.5 eV characteristic of pure silicon (Figure 2a, spectrum 1) [29]. The corresponding substrate was then oxidized under UV/O_3_ conditions. After oxidation, the high energy shift at 103.8 eV of the Si2p XPS spectrum was characteristic of the SiO_2_ layer (Figure 2a, spectrum 2) [29]. The thickness of the SiO_2_ layer obtained from XPS and ellipsometry measurements was about 6 nm. The oxidation was confirmed by the IR spectrum of the oxidized silicon surface (Figure 2b, spectrum 1) which exhibited the expected Si-O-Si vibrations at 1107 cm^−1^ [30]. In a second step, APTES surface modification was performed and confirmed by the appearance in the IR spectrum of two new vibration bands at 2850 and 2916 cm^−1^ (Figure 2b, spectrum 2). These signals corresponded to APTES symmetric and antisymmetric C-H stretching modes, respectively. An additional weak signal at 1450 cm^−1^ attributed to the scissoring NH vibration testified the presence of the added amine group [30]. Finally, in a third step, the coupling reaction between TbL and the APTES modified wafer was performed in DMF, in the presence of TBTU as a coupling agent and DIPEA as a base [25]. The IR spectrum of the corresponding TbL@Si wafer (Figure 2b, spectrum 3) exhibited additional vibrations in the range 1550–1650 cm^−1^ associated to the amide C = O vibration (1640 cm^−1^) and to the C = O vibrations of the immobilized TbL carboxylate groups (1570 cm^−1^). The IR spectrum also showed an increase in the intensities of the CH stretching bands as well as a newly formed absorption at 3400 cm^−1^ which can be attributed to the NH amide stretching mode.

**Figure 3:**
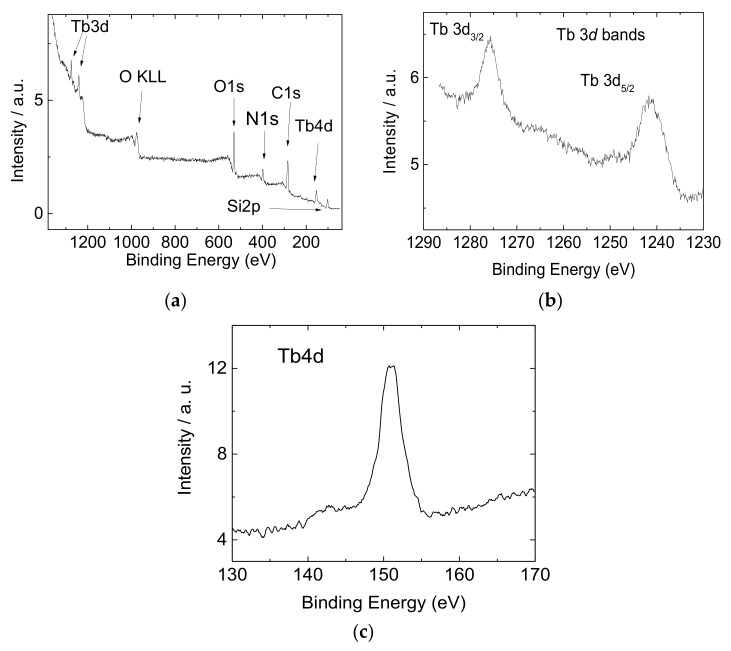
(**a**) Overall XPS spectrum of TbL@Si, (**b**) Tb 3d XPS spectrum of TbL@Si, (**c**) angle-resolved Tb 4d spectrum of TbL@Si.

Functionalized samples were also characterized by XPS measurements. A wide scan XPS survey of TbL@Si was performed to confirm the presence of Si, N, C, and O (Figure 3a) while the presence of Tb was confirmed at high binding energies by the Tb 3d level, which was split into two peaks (Tb 3d_5/2_ and Tb 3d_3/2_ at 1242.2 eV and 1276.2 eV, respectively) due to spin orbit coupling (Figure 3b).

In order to ensure that the XPS signal around 150 eV corresponded to the Tb 4d level and not to the Si 2s level, angle-resolved XPS measurements were performed. In these conditions it was possible to detect only the Tb 4d signal at 151 eV (Figure 3c), confirming the Tb oxidation state (Tb(III)) [31,32]. Finally, the thickness of the Tb grafted layer was determined by XPS and ellipsometry to be around 2.5 nm. This thickness was compatible with the formation of a single monolayer of TbL at the SiO_2_ surface. Indeed, if one first considered the crystal structure of a related Tb complex [33], the distance between two oxygen atoms borne by two opposite carboxylate arms of the ligand is about 1.2 nm, and second, the length of SiOSiCH_2_CH_2_CH_2_N linker is about 1 nm [34]. Therefore, this corresponded to a 2.2 nm distance at the SiO_2_ surface, which is, regarding the uncertainty of the measurements, close to the 2.5 nm determined for the thickness of the Tb grafted layer.

### 2.3. Luminescent Properties of the Functionalized Silicon Wafers

The optical properties of TbL@Si wafer were investigated by fluorescence experiments. The luminescence spectrum of TbL@Si wafer (Figure S4) exhibited a set of four signals between 450 nm and 650 nm that corresponded to the characteristic emission lines of Tb(III).

It is interesting to note that for TbL@Si, the terbium peak positions as well as the ratio intensities between these peaks were the same as those for the TbL complex in solution or in the solid state (Figure S4). This point indicated that the optical properties of the Tb(III) complex were not modified when the complex was grafted at the wafer surface. On the other hand, the fluorescence lifetime of TbL was reduced from 2.20 ms in the solid state to 1.33 ms for TbL@Si wafer. The presence of extra OH vibrators from the surface silanol groups which are coupling with terbium ions could lead to a partial non-radiative deactivation of the Tb(III) ^5^D_4_ excited state, leading to such a fluorescence lifetime decrease. Similar phenomenon was previously observed for Tb(III) in Tb exchanged MFI zeolites [35], or in fluorescent silica nanoparticles modified chemically with Tb complexes [36].

**Figure 4:**
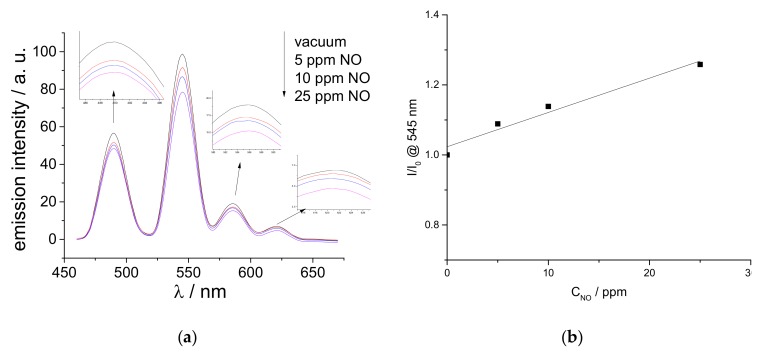
(**a**) Emission spectra of TbL@Si wafer exposed to various concentrations of gaseous NO at 298 K, λ_exc_ = 230 nm, (**b**) Stern–Volmer plot for the data, measured at an emission energy of 545 nm.

Preliminary fluorescence sensing experiments were conducted after the exposure of TbL@Si wafer to NO in the gas phase. For that, TbL@Si wafer was introduced into a homemade hermetic cell that was filled with the gas and its fluorescent response was recorded according to three selected NO concentrations (Figure 4).

Upon exposure to NO, the emission intensity from TbL@Si was reduced, the equilibrium value being reached a few minutes after NO introduction in the cell. After a vacuum cycle and NO removal, the initial emission spectrum was quickly recovered which highlighted the reversibility of the system (Figure S5). When NO concentrations of 5, 10, and 25 ppm were introduced in the cell, the intensity of the Tb(III) ^5^D_4_ to ^7^F_5_ emission line (545 nm) decreased by 8%, 12% and 21%, respectively (Figure 4a), and this evolution was linear according to NO concentration (Figure 4b). The linearity of the quenching percentage as a function of the quencher concentration can attest to an intramolecular deactivation of the TbL excited state in the presence of NO [37]. The Stern–Volmer (SV) quenching constant of the TBL@Si is around 0.01 ppm^−1^ which is in the same order of magnitude of the SV constant (0.02 ppm^−1^) obtained by the pioneered work of Sailor et al. [12] for solid-state sensors. Finally, the relative intensities of the ^5^D_4_ to ^7^F_J_ transitions for TbL@Si wafer could be indicative of the underlying quenching mechanism. Indeed, as the relative intensities of the ^5^D_4_ to ^7^F_J_ transitions are sensitive to the Tb(III) environment [38], a change in the Tb(III) coordination sphere is expected to induce a modification of the emission band intensity ratios, viz I_490_/I_545_, I_585_/I_545_, and I_622_/I_545_ ratios. Here, when TbL@Si wafer was exposed to various amounts of NO, these ratios remained unchanged (I_490_/I_545_ around 60%, I_585_/I_545_ around 19% and I_622_/I_545_ around 7%, respectively, Figure S6). Consequently, the luminescence quenching did not seem to be driven by direct insertion of NO in Tb(III) coordination sphere but triggered by the non-radiative energy transfer from the ^5^D_4_ excited state of the probe to the NO vibrator at the proximity of TbL@Si surface.

## 3. Materials and Methods

### 3.1. Reagents

All chemicals and solvents were used as supplied from commercial sources. The lanthanide salts and cyclen were purchased from Strem Chemicals, Newburyport, MA, USA. All other chemicals were supplied by Sigma Aldrich, St. Louis, MO, USA or Alfa Aesar, Haverhill, MA, USA. Silicon wafer (110) was supplied by Siltronix, Archamps, France.

### 3.2. Instrumentation

All ^1^H and ^13^C NMR experiments were carried out using a 500 MHz Bruker Avance II NMR spectrometer or a 250 MHz Bruker Avance I NMR spectrometer (Bruker, Billerica, MA, USA). Infrared (IR) absorption spectra for ligands and complexes were recorded using a PerkinElmer Spectrum Two IR spectrometer (Perkin Elmer Waltham, MA, USA). IR absorption spectra for silicon wafers were recorded with a Bruker Vertex 70 spectrometer (Bruker, Billerica, MA, USA). Mass spectrometry experiments for ligands and complexes were performed on a Micromass Q-TOF electrospray mass spectrometer (Micromass, Manchester, UK).

### 3.3. X-Ray Photoelectron Spectroscopy

XPS spectra were measured using the non-monochromatized AlKα radiation at 1486.6 eV. The photoelectrons were collected by a Scienta SES 200 hemispherical analyser (Scienta, Taunusstein, Germany). The overall resolution of the setup was about 0.8 eV. Spectra from the C 1s, O 1s, N 1s, Si 2p, Tb 4d, and Tb 3d bands were systematically recorded as no other elements than the previous ones were detected. For each sample, at least five different locations were investigated to ensure the homogeneity of the surface. All XPS spectra were corrected for any charging effects by fixing the C 1s binding energy at 284.8 eV and were treated with Shirley background subtraction. XPS measurements were also performed following standard procedures to determine the thickness of the different layers [39,40]. These thicknesses measurements were also checked by ellipsometry using an UVISEL ellipsometer from Horiba, Kyoto, Japan using published procedure [41].

### 3.4. Fluorescence Spectroscopy and Gas Dilution

Fluorescence measurements were carried out using a Varian Cary Eclipse fluorescence spectrophotometer (Agilent, Santa Clara, CA, USA). For the gas sensing experiments, a homemade setup for performing ppm NO concentrations inside the spectrofluorometer was developed. For that, a customized fluorescence cell that allowed gas filling at known concentrations was designed. Various concentrations were obtained by diluted gas from etalon bottle with pure nitrogen, as it was checked that pure nitrogen does not have any effect on the fluorescence intensity. The dilution was carried out by mass flowmeter in a special mixing chamber to perform homogenous mixture. To produce the ppm concentrations, two consecutive dilutions were needed. The first one, to produce a 100 ppm mixture at high pressure (up to 5 bars), and the second one, from the former 100 ppm one, to produce a lower concentration mixture. To ensure the reproducibility of the experiments, all pipes and intermediate tank were drained to a secondary vacuum. The gas at the desired concentration was used to fill a chamber connected to the fluorescence cell containing TbL@Si wafer, up to atmospheric pressure. The cell was finally sealed and used for fluorescence quenching experiments.

### 3.5. Synthesis of TbL

All the organic compounds were prepared according to reported procedures [24,25]. 2-(4,7,10-tris(carboxymethyl)-1,4,7,10-tetraazacyclododecan-1-yl) pentanedioic acid DOTAGA.HCl.4H_2_O, 293 mg, 0.501 mmol) was dissolved in deionized water (10 mL). Terbium oxide (100 mg, 0.273 mmol) was then added to the solution. The reaction mixture was heated to 95 °C for 24 h. The solution was cooled to room temperature and the pH was increased to 10 to remove the excess Tb(III) by precipitating it as Tb(OH)_3_. The precipitate was removed and the solution was evaporated to dryness to get the product as an off-white powder.

TbL: Yield = 80%. IR ν(CO) = 1724 cm^−1^ (free COOH), 1594 cm^−1^ (coordinated COO^−^). Elemental Analysis, calculated for C_19_H_27_Na_2_N_4_O_10_Tb(H_2_O)_5_(NaCl): C, 27.67; H, 4.52; N, 6.79. Found C, 27.46; H, 4.39; N, 6.74. HRESI-TOF: calculated for (C_19_H_28_N_4_O_10_Tb)^−^ = 631.1059 − found 631.1058, calculated for (C_19_H_27_N_4_NaO_10_Tb)^−^ = 653.0878 − found 653.0877.

### 3.6. Preparation of Silicon Wafers

Silicon wafers were cut into 1 cm^2^ squares, treated with HF buffer for 5 minutes to remove the native oxide layer, then washed with water, dried and finally subjected to UV/O_3_ oxidation.

### 3.7. Silicon Wafer Functionalization

The silicon wafer was immersed into a toluene solution (16 mL) containing APTES ((3-aminopropyl)triethoxysilane, 4 mL) and the solution was stirred for 24 h with occasional sonication. It was then washed with acetone, sonicated, and dried. The APTES modified wafer was then introduced into a DMF solution (10 mL) containing 13 mg of TbL (0.016 mmol, 1eq), 27 µL of DIPEA (0.155 mmol, 10 eq) and 25 mg of TBTU (0.078 mmol, 5 eq). The solution was stirred for 24 h with occasional sonication and afterwards the TbL functionalized wafer, TbL@Si was washed with ethanol and acetone, dried and used for characterization and studies.

## 4. Conclusions

To sum up, a Tb-DOTAGA (TbL) complex was synthesized and grafted onto an amine-modified silicon wafer (TbL@Si). The strategy implied control of the functionalized Si wafer which was followed by means of IR absorption spectroscopy, ellipsometry, and XPS experiments. The optical properties of TbL@Si were examined by photoluminescence experiments. The fluorescence of TbL at the surface of the wafer was similar to fluorescence of TbL in the solid state, except from the point of view of the fluorescence lifetime. Indeed, for the grafted complex, the fluorescence lifetime decrease could be interpreted by the influence of surface silanol groups coupling with Tb(III) ions, leading to a partial non-radiative deactivation of the Tb(III) ^5^D_4_ excited state. The ability of TbL@Si wafer to sense NO in the gas phase was tested and the results showed that the fluorescent response of TbL@Si wafer was partially quenched according to increasing NO concentrations. Moreover, the initial fluorescence of the sample was recovered as soon as NO was removed, showing the reversibility of the response. Our approach offers the possibility of performing toxic NO gas sensing at a very low level of 1 ppm. It can be assumed that the present method might be adapted for other lanthanides and to sense different gases.

## Supplementary Materials

The following are available online at https://www.mdpi.com/1420-3049/24/10/1914/s1, Figure S1: IR spectra of DOTAGA (L) and TbDOTAGA (TbL), Figure S2: High Resolution ESI MS spectrum of TbL (negative mode, MeOH), Figure S3: Determination of q, the number of water molecules coordinated to the Tb(III) center, Figure S4: Emission spectra of TbL (TbDOTAGA) in aqueous solution, solid state, and grafted at the Si surface (TbL@Si), Figure S5: TbL@Si 545 nm emission band evolution on the course of vacuum/NO cycles, Figure S6: Evolution of the relative emission band intensities according to the NO concentration, the reference being the 545 nm emission (λ_exc_ = 230 nm, black—490nm, light grey—545 nm, grey—585 nm, dark grey—622nm).
