# Supplementary material for: Silicon Wafer Functionalization with a Luminescent Tb(III) Coordination Complex: Synthesis, Characterization, and Application to the Optical Detection of NO in the Gas Phase
Source: Molecules. 2019 May 17;24(10):1914. doi: 10.3390/molecules24101914 (PMC6572424; doi:10.3390/molecules24101914)
Supplement: Supplementary file 1 [file molecules-24-01914-s001.pdf]

"Silicon wafer functionalization with a luminescent Tb(III) coordination complex: synthesis, characterization and application to the optical detection of NO in the gas phase"

Dr Bijal Kottukkal Bahuleyan, K. Toussaint *et al.*

Supplementary Materials

Contents

Figure S1: IR spectra of DOTAGA (L) and TbDOTAGA (TbL)

Figure S2: High Resolution ESI MS spectrum of TbL (negative mode, MeOH).

Figure S3: Determination of q, the number of water molecules coordinated to the Tb(III) center

Figure S4: Emission spectra of TbL (TbDOTAGA) in aqueous solution, solid state and grafted at the Si surface (TbL@Si)

Figure S5: TbL@Si 545 nm emission band evolution on the course of vacuum / NO cycles.

Figure S6: Evolution of the relative emission band intensities according to the NO concentration, the reference being the 545 nm emission (λ_{exc} = 230 nm, black - 490nm, light grey - 545 nm, grey - 585 nm, dark grey – 622nm).

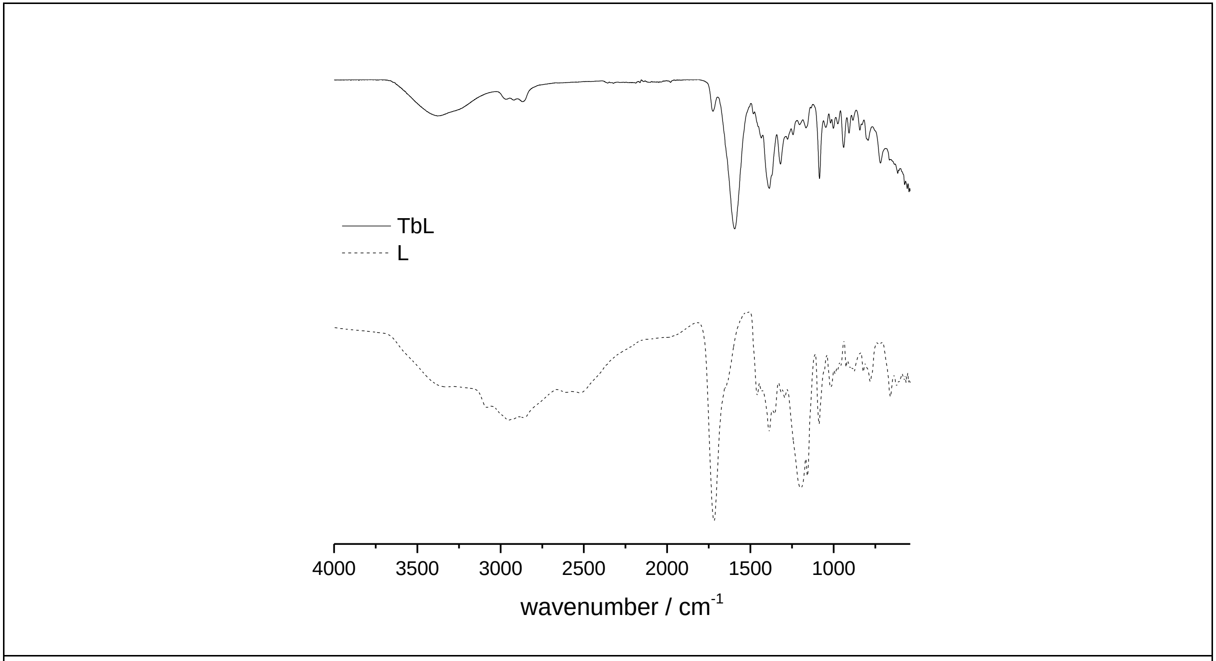

Figure S1. IR spectra of DOTAGA (L) and TbDOTAGA (TbL)

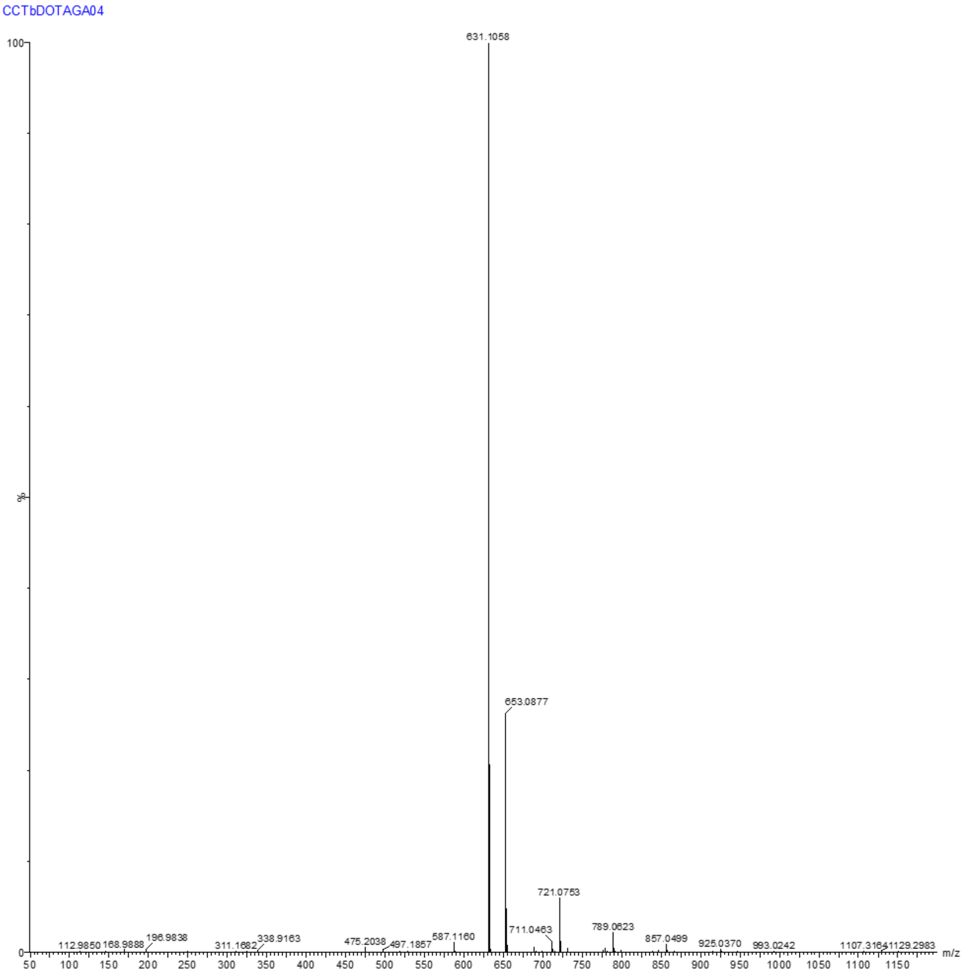

Figure S2. High Resolution ESI MS spectrum of TbL (negative mode, MeOH).

Figure S3: Determination of q, the number of water molecules coordinated to the Tb(III) center

In order to determine the number of water molecules present in the lanthanide inner coordination sphere, luminescence lifetime measurements of TbL complex in H_2O/D_2O mixtures were performed (0 < H_2O molar fraction < 1). The calculation of the number of metal-bound water molecules (q) was possible, owing to the well-established isotope effect [28] and by using the empirical relationship derived from the Horrocks and Sudnick equation q = 4.86 $(\frac{1}{\tau_{H2O}} - \frac{1}{\tau_{D2O}})$ with $\tau_{H20} = 2.09\, ms$ and $\tau_{D20} = 3.58\, ms$. This leads to a value of 0.96 for q, *i.e.* 1 water molecule coordinated to the Tb center.

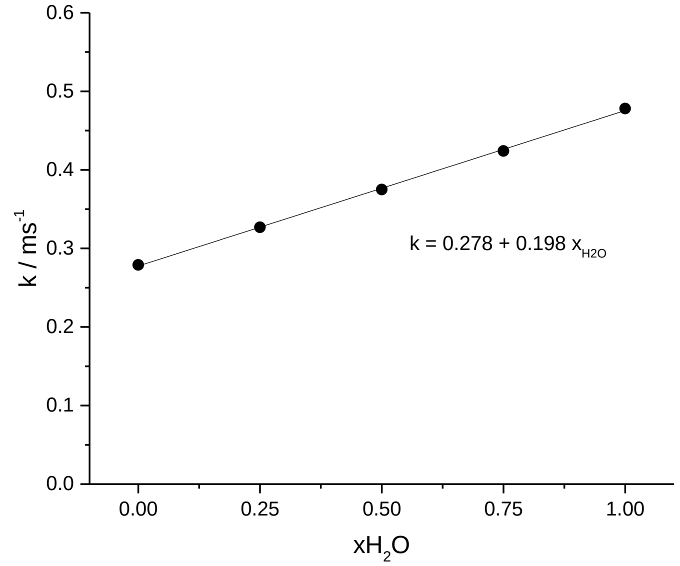

Figure S3. Plot of the Tb(III) luminescence decay rate constants (k = $\frac{1}{\tau}$) against the molar fraction of H_2O in H_2O/D_2O mixtures (C_{TbL} = 6 mM).

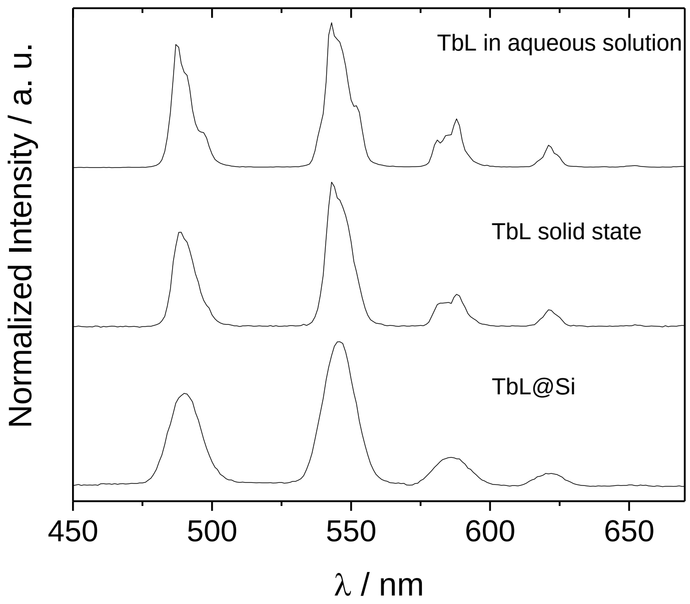

Figure S4. Emission spectra of TbL (TbDOTAGA) in aqueous solution, solid state and grafted at the Si surface **(**TbL@Si**)** (λ_{exc} = 230 nm).

The lowering of the resolution for the emission of TbL@Si is due to the fact that the slit have to be more opened to observe the luminescence of the monolayer compared to the pure complex in solution or in the solid state.

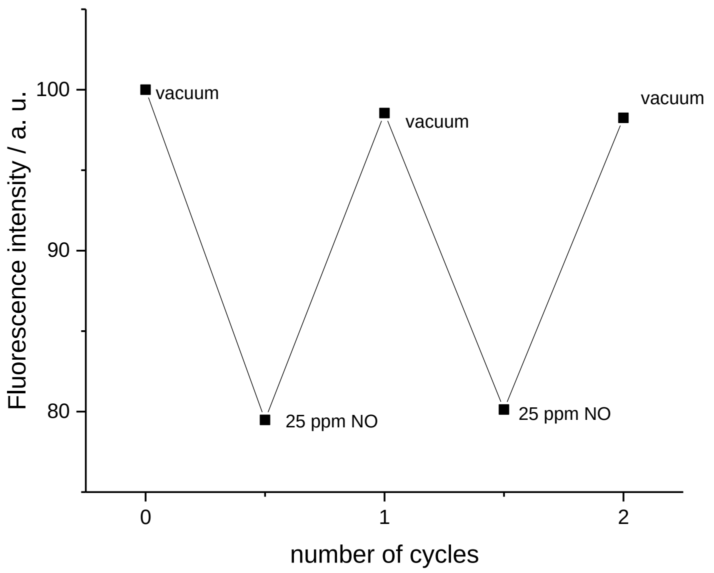

Figure S5. TbL@Si 545 nm emission band evolution on the course of vacuum / NO cycles.

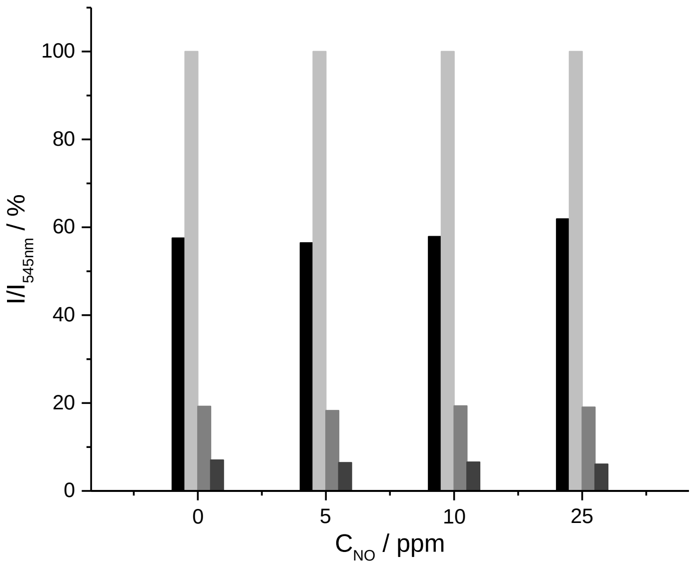

Figure S6. Evolution of the relative emission band intensities according to the NO concentration, the reference being the 545 nm emission (λ_{exc} = 230 nm, black - 490nm, light grey - 545 nm, grey - 585 nm, dark grey – 622nm).
